# Simulated learning in rural community environment: pushing the boundary

**DOI:** 10.1186/s41077-021-00155-3

**Published:** 2021-02-17

**Authors:** Burhanuddin Ali Akber, Mehak Ismail Rajani, Farah Khalid, Charles Docherty

**Affiliations:** Centre for Innovation in Medical Education, The Aga Khan University, National Stadium Road, Karachi, Sindh 74800 Pakistan

**Keywords:** Simulation-based education, Simulation specialists, Standardized patients, Community healthcare

## Abstract

Experiential learning through simulation can play a very significant role, not only in hospital settings but also in community contexts (Lubbers and Rossman, Nurse Educ. Today 48:140-144; Wheeler and McNelis, Nurs. Educ. Perspect 35:259-261). This paper discusses the concept of creating a novel simulated village set-up within a modern simulation center, to effectively deliver contemporary learning outcomes. It also highlights the challenges and risks of developing a simulated village set-up and strategies to counteract them. Furthermore, it describes the role of simulation specialists as innovators and explicates the gamut of expertise in education, management, and technologies that are required to deliver excellence in simulation-based education.

## The need for a simulated community environment

Aga Khan University’s (AKU) Centre for Innovation in Medical Education (CIME), Pakistan, a recent Society for Simulation in Healthcare (SSH)-accredited simulation center, comprises 80,000 ft^2^ of mostly simulated hospital environments for the education of medical and nursing students, and healthcare professionals from across the country [1]. With stability and growing affluence comes increased life expectancy and a growing middle class in Pakistan, and a double-whammy of existing communicable and a tsunami of non-communicable diseases [2]. Community healthcare is of growing importance, with doctors and nurses striving to improve the health and well-being of communities through education in disease prevention, safe health practices, nutrition, and wellness, and challenging the more traditional healthcare beliefs that confound modern medicine [3]. Assisted living is becoming more important for a growing number of those living with a compromised ability to perform the activities of living unaided [4]. Although the demographic profile of Pakistan points to a predominantly youthful population [5], the growing number of elderly people with concomitant chronic cardiovascular, respiratory, and neurological conditions require a re-focusing of health services along with re-balancing of the education and continuous development of healthcare professionals [2, 6]. Similarily, the pediatric population of the country is also deprived of access to quality healthcare which is evident through alarming mortality statistics quoted in the Pakistan demographic health survey 2017–2018 (PDHS). It reports that childhood mortality rates are higher in rural areas than in urban areas by 10 deaths per 1000 live births. Neonatal, infant, and under-5 mortality rates are 45, 68, and 83 deaths per 1000 live births, respectively, in rural areas, as compared with 37, 50, and 56 deaths per 1000 live births in urban areas [7].

These statistics indicate poor healthcare services in rural community settings, hence the dire need for training the healthcare professionals working in these areas.

Simulation-based education is of proven worth in hospital settings but what about the community? Most hospitals look and feel very similar, being based upon the universally accepted “medical model” of care. There is no “template” for community learning spaces, as models of community care are very much culturally bound and locally determined by issues such as multi-generational living, affluence, and poverty [8, 9]. Within Pakistan, the sixth most populous country with 61% of the people residing in rural areas, extremes of wealth and poverty mean that vast differences in community environments exist [10].

**Fig. 1:**
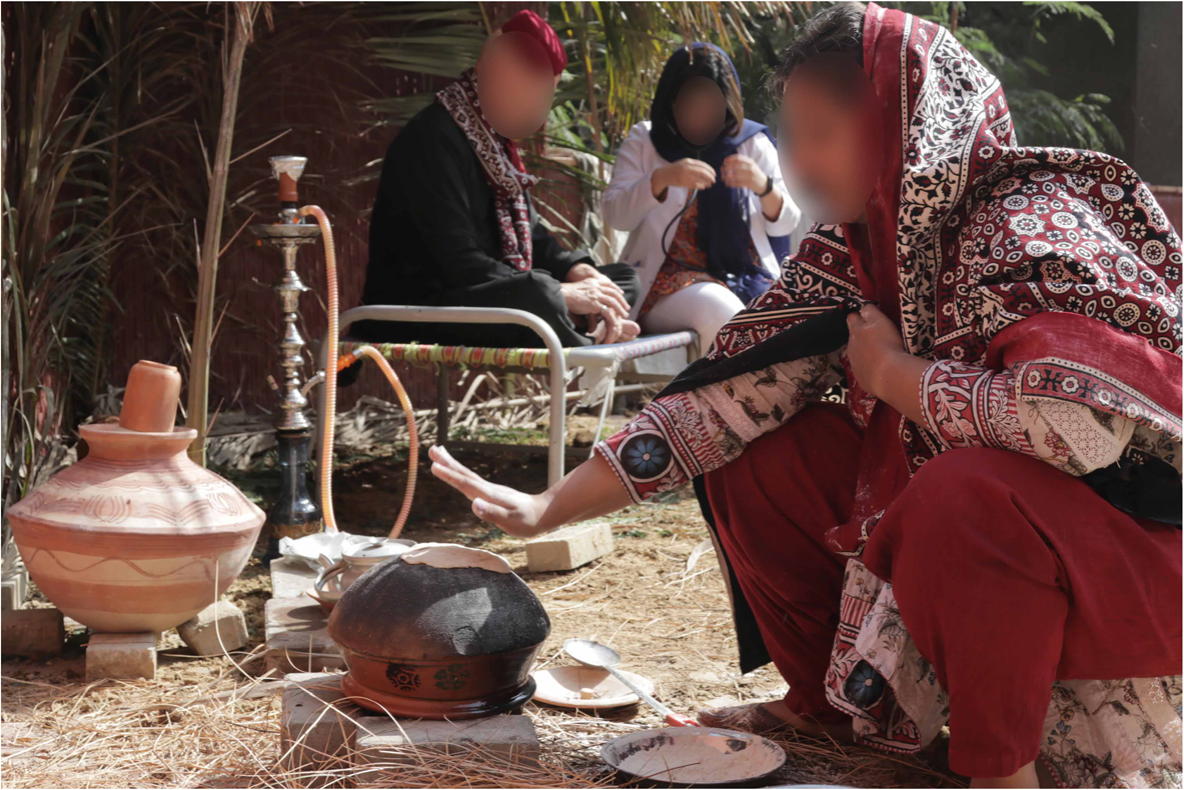
Examples of village setup

**Fig. 2:**
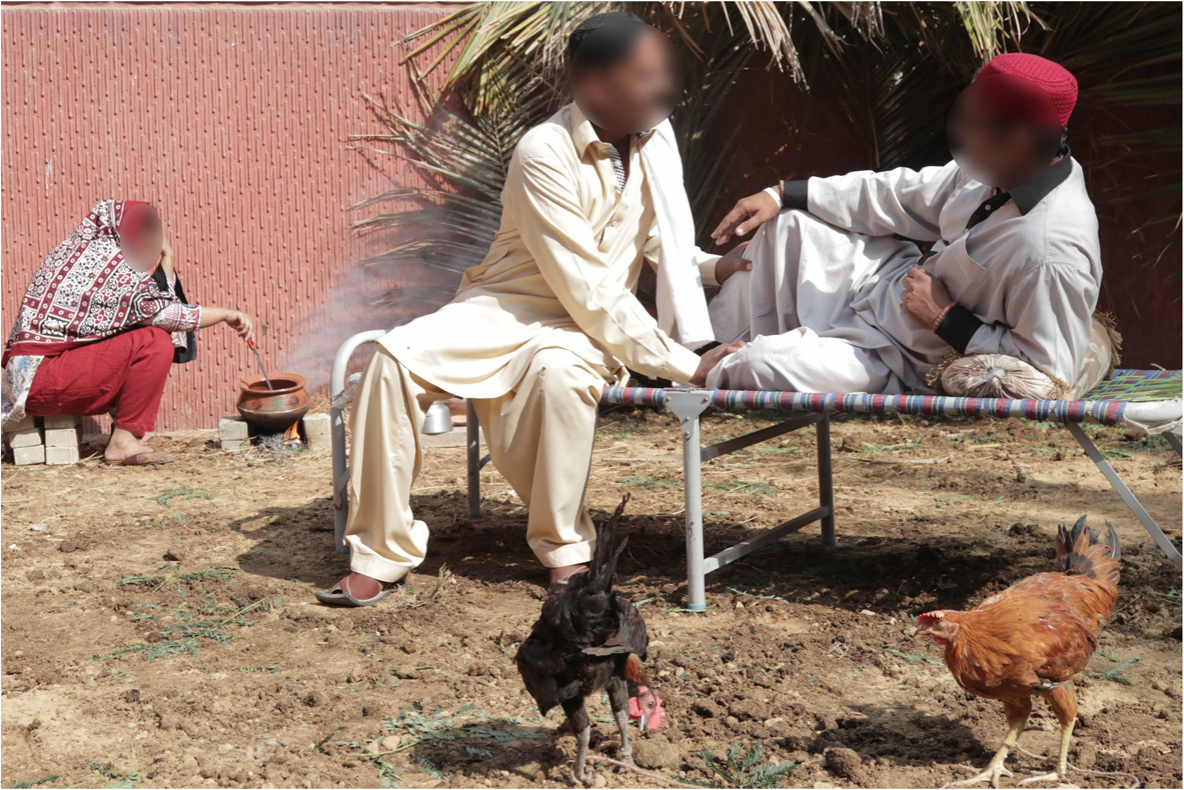
Examples of village setup

Simulation-based education provides a means of achieving experiential learning. It helps the learners to use their existing knowledge by linking it with new experiences [11]. For this reason, CIME has recently added two simulated community environments, one depicting a more westernized middle-class environment with a bedroom, kitchen, and bathroom and the other being an outdoor typical village or rural space that can be recreated and tailored as required. As a new development in our center, our stakeholders with community foci are beginning to use our “middle class” community flat, which is indoors ad air-conditioned, in many ways is easier on both staff and students for active learning. However, the center has had to encourage groups who could exploit the rural, village set-up, by actively marketing its potential on the center’s web pages, Facebook, and Instagram accounts (Figs. 1 and 2).

The first group to use this for teaching purposes was from AKU’s post-RN BScN program who had received an internal AKU grant awarded by the Scholarship of Teaching and Learning (SOTL) to incorporate simulation. This provided an ideal opportunity for faculty to work with the center’s team to construct, install technology, and facilitate learning, thus testing the concept and practicalities of using such a space for simulated practice.

Acknowledging that conducting effective clinical simulation experiences requires expertise in many domains, this article is written from the perspective of simulation specialists, whose innovation and expertise in the technical and organizational support of the educational process often goes unnoticed. These simulation specialists are working in the capacity of technical and operational management and have been serving the center for around 4 years. They have acquired training in simulation programming nationally and internationally. Also, they are eligible and are in the process of taking examination for the Certified Healthcare Simulation Operations Specialist (CHSOS) certification.

There is limited evidence regarding teaching community health nursing using simulation in rural settings. In Pakistan, such on-campus learning is appealing, not least because security and transportation issues make community learning logistically problematic. While there are only a few studies reporting the engagement of standardized patients (SPs) in community settings [12], using a campus-based setting facilitates their use more easily. Neither are there reports in the literature narrating the crucial facilitating role of simulation specialist staff in driving curricular innovation, but as their growing professionalization demands, and as their power over technology and expert authority increase, comes the responsibility to disseminate achievements and experiences to a wider audience.

This paper discusses the strategies and preparation required for the recreation of a simulated village area in the Aga Khan University, Centre for Innovation in Medical Education (AKU, CIME), to provide students with the opportunity of achieving community-based learning outcomes. The paper further highlights the phases of organizing such events from the perspective of the center, and its role in organizing the ways, means, materials, and technologies and providing expertise and guidance in the recreation of a village space that serves as a suitable learning environment for students where the simulation process can occur.

## Planning and development

### Learning goals

It is often reported by the students that they feel unacquainted during their community home visit. However, they are anticipated to conduct and execute assessments and diagnosis of individuals, and groups belonging to the communities and provide continuum of health care. They are also trained to identify high-risk groups. The learning goal of this village set-up is to help the students to learn from their own mistakes in a safe space before they are subjected to an actual community setting. The outcome of this activity is to provide confidence to the students and enhance their communication skills as they learn how to negotiate during challenging situation. The simulated rural community environment aims to provide an optimal learning to its participants by providing real-life experience of a village in a developing country such as Pakistan.

### Site identification

The center’s village setup is an open area with walls at two sides and scrubland at the other, with a watercourse overshadowed by trees ideally representing many habitats in the countryside surrounding the big cities such as Karachi. It is spacious enough to accommodate multiple sections of a village, representing the typical living conditions of its residents. To transform this space, a list of essential materials, equipment, and props were identified to complete the picture of a homely setting and to achieve the level of fidelity required to help students suspend their disbelief and to achieve the maximum transfer of learning.

### Resources for setting up the environment

**Fig. 3:**
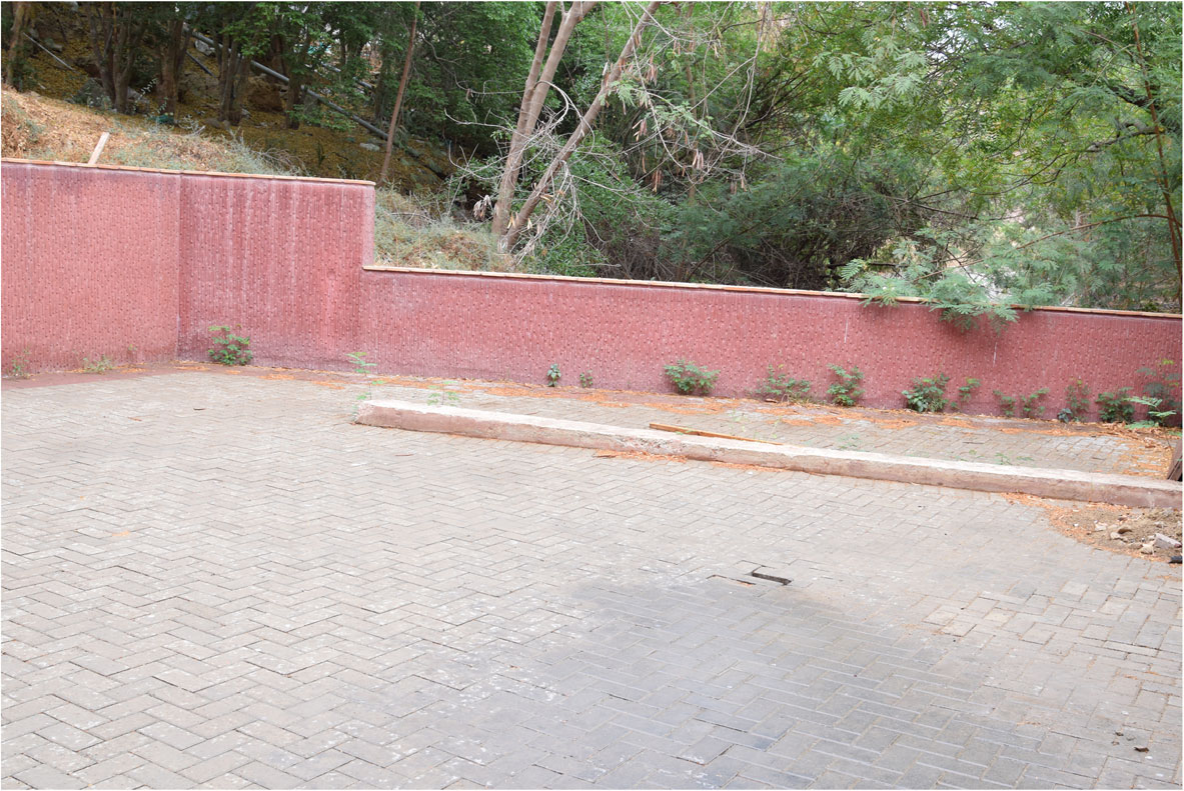
The basic set-up of the village environment

**Fig. 4:**
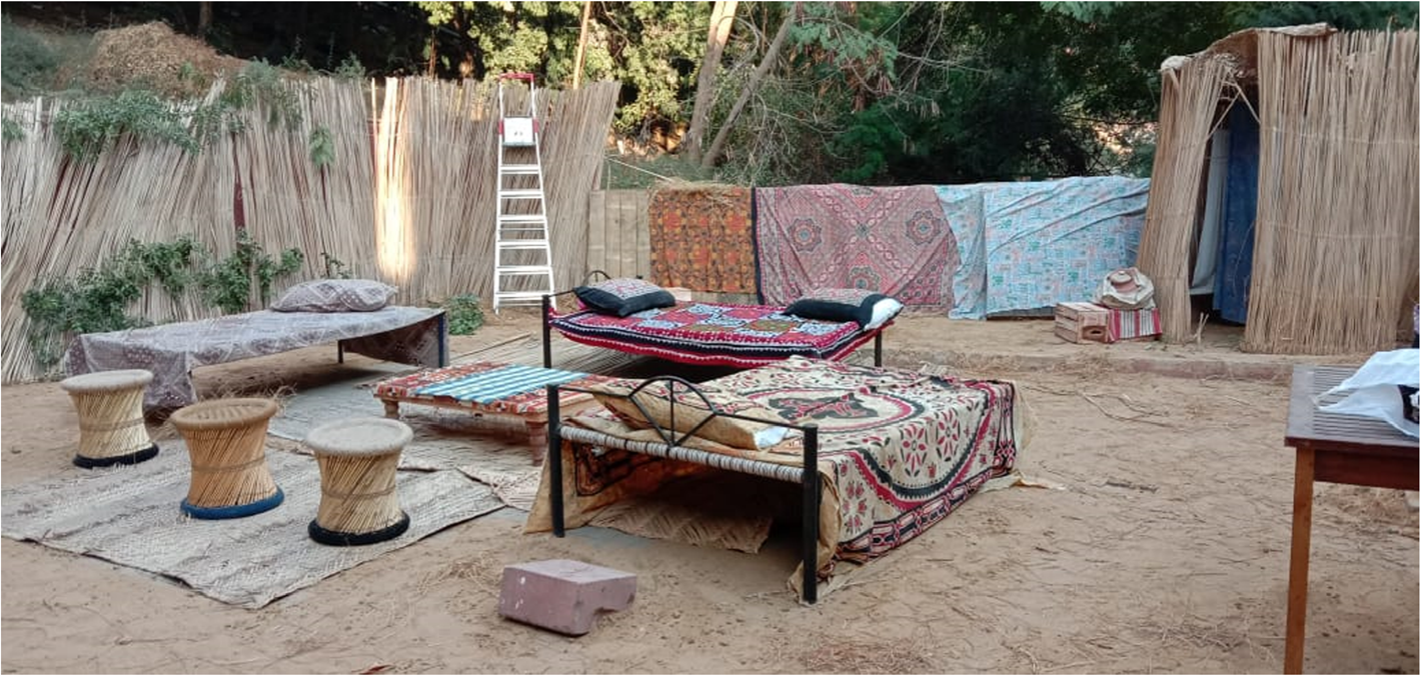
The basic set-up of the village environment

The basic set-up of the village environment includes disguising the walls with bamboo sheets and floor covered with sand and straw (Figs. 3 and 4). This landscape can be further modified according to different simulated scenarios’ requirements. “Charpoy”, a multipurpose piece of furniture used by villagers for resting, dining, and casual discussions, was strategically placed and strewn with hand-crafted traditionally designed blankets and pillows. To facilitate village meetings and discussions between community health workers and villagers, a centrally focused area was furnished with bamboo chairs, wooden tables, and mats to create the sitting area. Smoking “hookah” is one of the recreational activities practiced by villagers and was carefully placed. This specific setup was used in a scenario where community health nurses negotiated with the community stakeholders to prioritize local health-related issues. It was a part of the community health nursing grant, funded by SOTL. Likewise, for scenarios requiring a home setting in the community, bamboo sheets can be rearranged to replicate a typical house and charpoy can be placed to be used as a bed for the patient.

### Technical requirement

It is now fairly standard to facilitate the debriefing process through technology. A B-Line portable system is utilized for video capturing and recording of the simulation which can be later used for video-stimulated recall during the debriefing of participants. AV-IT resources such as a laptop to operate the B-Line debriefing system, lapel and cordless mics to record conversations, digital mixers, speakers, and extension boards are used to cover the overall technology needs. All of these are backed and operated by our center’s technical team who are experts in their respective fields.

### Use of standardized patients

**Fig. 5:**
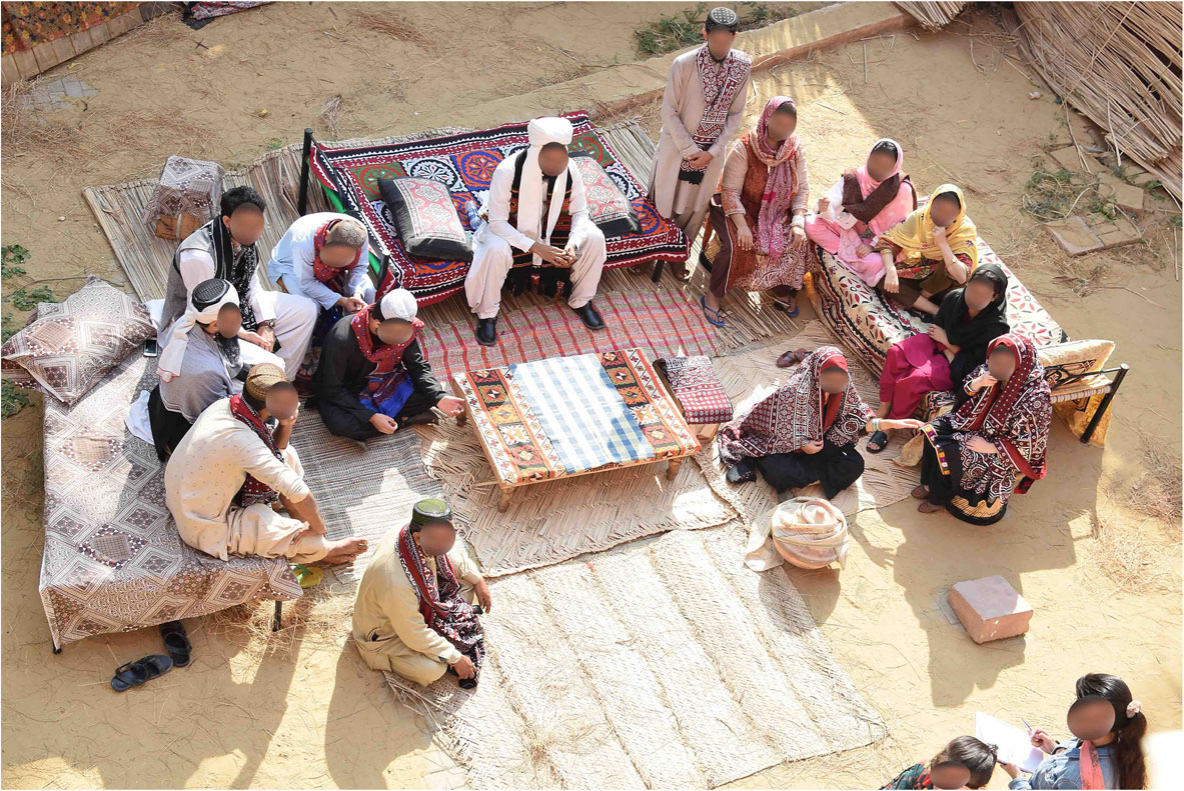
Traditional attire completed the colorful picture of the villagers

SPs are used as locals in the simulated scenarios, playing the roles of stakeholders (Religious leader (Molvi), Community-based organization leader (Wadera), School Principal/Master, Health care representative (UC Nazim), and a Local Health Centre Nurse) or as patients requiring medical treatment. These SPs acting as community leaders are dressed up in appropriate garb, and head-dress, providing an authentic visual demonstration of authority, which is crucial to adding fidelity to the scenario. Also traditional attire such as ajrak shawls, traditional caps, false beards, and mustaches completed the colorful picture of the villagers (Fig. 5).

### Use of simulators

Demonstrating a lady in labor in a home environment can be easily done by using high-fidelity birthing simulator on a charpoy. This setting is very close to reality and can challenge the students to manage such situations in resource scarcity. Similarly, scenarios dealing with an adult or pediatric health can be replicated using full-body high-fidelity simulators. An example can be the resuscitation of an adult who has collapsed on an uneven floor and in a congested home environment.

## Limitations and recommendations

### Environment

After running the scenario with community health nursing, a few limitations of the setup were identified. With respect to the environment, sound control was most difficult: there was a nearby dining outlet that had created a distraction, with sounds that were incongruous to the setting and threatened fidelity. This can be prevented through looping a pre-recorded background track of village sounds—hens, chickens, motorbikes, and children at play—through portable speakers.

### Technical

The absence of sufficient shade was a potential risk to the sensitive equipment placed in the open area. This can be mitigated by adding shades or by operating these equipment within a cool shaded area adjacent to the scenario setting. Also for the safety of equipment from both heat and the risk of rain or dust storms, daily set-up and dismantling are preferred.

### Standardized patients

During the initial simulation, the weather was bearable; however, as the day progressed, the scorching sunlight made SPs jobs difficult, who performed without shelter throughout the day. It was also noted that the SPs used to give in easily during the arguments and were not flexible enough to adjust as the simulation evolved. This limitation can be mitigated in the future with rigorous and more thorough training of SPs. Additionally, using confederate mic and headset, in-time instructions can be given to manage the simulation better.

### Simulators

As is standard “best practice” before the day of activity, a short “dry run” or “pilot” is usually planned with SPs, support staff, simulation educators, and stakeholders to identify changes in the scenario if required. This is helpful in advance troubleshooting of any problems while operating simulators in an open area and also to measure the maximum range of distance between the simulator operating device and actual simulators.

## Conclusion and considerations

The first simulated rural community environment was created in CIME to teach the undergraduate nursing students about the community settings before their actual experience. As there were no previous examples for a simulated rural community setup, the innovative administrative and technical approach from a modern medical simulation center, to facilitate the formation of a real-life community setup, has ultimately provided the students an experience to practice their skills and gain confidence before being subjected to the actual environment. The experience of organizing such activity has helped the team in expanding their capacity and to initiate more of such novel ideas in simulation which are rarely capitalized. The center is also considering to utilize the village setup for simulation activities such as different complexities during child birth, mental health assessment, triage activity, assisted living, and ambulatory care. It is also planned that in future, the team would conduct multiple researches to gauge the impact of conducting simulated rural community scenarios for students learning.

## Data Availability

Not applicable

## Abbreviations

AKU: Aga Khan University
CIME: Center for Innovation in Medical Education
SP: Standardized patient
BScN: Bachelors of Sciences and Nursing
AV-IT: Audio visual-information technology
CHSOS: Certified Healthcare Simulation Operations Specialist
